# In Vitro Anti-Diabetic, Anti-Inflammatory, Antioxidant Activities and Toxicological Study of Optimized *Psychotria malayana* Jack Leaves Extract

**DOI:** 10.3390/ph16121692

**Published:** 2023-12-05

**Authors:** Sharifah Nurul Akilah Syed Mohamad, Alfi Khatib, Siti Zaiton Mat So’ad, Qamar Uddin Ahmed, Zalikha Ibrahim, Tanzina Sharmin Nipun, Humaryanto Humaryanto, Mohamed F. AlAjmi, Shaden A. M. Khalifa, Hesham R. El-Seedi

**Affiliations:** 1 Pharmacognosy Research Group, Department of Pharmaceutical Chemistry, Kulliyyah of Pharmacy, International Islamic University Malaysia, Kuantan 25200, Pahang Darul Makmur, Malaysia; schaqilah91@gmail.com (S.N.A.S.M.); dszaiton@iium.edu.my (S.Z.M.S.); quahmed@iium.edu.my (Q.U.A.); zalikhaibrahim@iium.edu.my (Z.I.); 2 Central Research and Animal Facility, Kulliyyah of Science, International Islamic University Malaysia, Kuantan 25200, Pahang Darul Makmur, Malaysia; 3 Faculty of Pharmacy, Airlangga University, Surabaya 60155, Indonesia; 4 Department of Pharmacy, Faculty of Biological Sciences, University of Chittagong, Chittagong 4331, Bangladesh; tnp99@gmail.com; 5 Faculty of Medicine, Universitas Jambi, Jambi 36122, Indonesia; humaryanto_fkik@unja.ac.id; 6 Department of Pharmacognosy, College of Pharmacy, King Saud University, P.O. Box 2457, Riyadh 11451, Saudi Arabia; malajmii@ksu.edu.sa; 7 Psychiatry and Psychology Department, Capio Saint Göran’s Hospital, Sankt Göransplan 1, 112 19 Stockholm, Sweden; shaden.khalifa@regionstockholm.se; 8 International Research Center for Food Nutrition and Safety, Jiangsu University, Zhenjiang 212013, China; 9 Department of Chemistry, Faculty of Science, Menoufia University, Shebin El-Kom 31100107, Egypt; 10 International Joint Research Laboratory of Intelligent Agriculture and Agri-Products Processing, Jiangsu Education Department, Jiangsu University, Nanjing 210024, China

**Keywords:** *Psychotria malayana* Jack, anti-diabetic, anti-inflammatory, antioxidant, toxicity, GC-MS, LC-MS

## Abstract

*Psychotria malayana* Jack (Family: Rubiaceae, local name: Salung) is a traditional herb used to treat diabetes. A previous study by our research group demonstrated that *P. malayana* methanolic and water extract exhibits significant potential as an effective agent for managing diabetes. Further research has been performed on the extraction optimization of this plant to enhance its inhibitory activity against α-glucosidase, a key enzyme associated with diabetes, and to reduce its toxicity. The objectives of this study are to evaluate the anti-diabetic, anti-inflammatory, and antioxidant properties of the optimized *P. malayana* leaf extract (OE), to evaluate its toxicity using a zebrafish embryo/larvae model, and to analyze its metabolites. The anti-diabetic effects were assessed by investigating α-glucosidase inhibition (AGI), while the inflammation inhibitory activity was performed using the soybean lipoxygenase inhibitory (SLOXI) test. The assessment of antioxidant activity was performed utilizing FRAP and DPPH assays. The toxicology study was conducted using the zebrafish embryo/larvae (*Danio rerio*) model. The metabolites present in the extracts were analyzed using GC-MS and LC-MS. OE demonstrated significant AGI and SLOXI activities, represented as 2.02 and 4.92 µg/mL for IC_50_ values, respectively. It exhibited potent antioxidant activities as determined by IC_50_ values of 13.08 µg/mL (using the DPPH assay) and 95.44 mmol TE/mg DW (using the FRAP assay), and also demonstrated an LC_50_ value of 224.29 µg/mL, which surpasses its therapeutic index of 111.03. OE exhibited a higher therapeutic index compared to that of the methanol extract (13.84) stated in the previous state of the art. This suggests that OE exhibits a lower level of toxicity, making it safer for use, and has the potential to be highly effective in its anti-diabetic activity. Liquid chromatography–mass spectrometry (LC-MS) and gas chromatography–mass spectrometry (GC-MS) demonstrated the presence of several constituents in this extract. Among them, several compounds, such as propanoic acid, succinic acid, D-tagatose, myo-inositol, isorhamnetin, moracin M-3′-O-β-D-glucopyranoside, procyanidin B3, and leucopelargonidin, have been reported as possessing anti-diabetic and antioxidant activities. This finding offers great potential for future research in diabetes treatment.

## 1. Introduction

Diabetes is a devastating chronic disorder that is caused by a cascade of events manifested by the decrease in or the blockage of insulin release from the pancreas. Ineffective insulin production or use causes hyperglycemia, or high blood glucose, which leads to organ and tissue damage over time [1]. About 422 million people worldwide have diabetes, most in low- and middle-income countries, and 1.5 million die from it each year. The number and prevalence of people with diabetes have been rising for decades [2]. The global incidence of diabetes is on the rise, where 537 million persons in the age range of 20–79 years have been diagnosed, constituting 10.5% of the total adult population within this age bracket. The global prevalence of diabetes is expected to reach 643 million individuals by the year 2030 and is anticipated to rise further to 783 million by 2045 [3]. According to the International Diabetes Federation [4], there has been a consistent upward trend in the prevalence of diabetes in South-East Asian countries over the past two decades. The latest estimates have surpassed all previous projections.

The increased prevalence of diabetes necessitates the use of medicinal herbs for both treatment and prevention. An increasing number of people are looking for ways to incorporate natural bioactive substances in alternative medicine due to their low toxicity, low cost, fewer side effects, and ease of acquisition [5]. One promising herb is *Psychotria malayana* Jack, known in Malaysia as “salung”. It is widely distributed over the areas of the Philippines, Indonesia, Malaysia, and Papua New Guinea [6]. *P. malayana* is traditionally used in the treatment of diabetes, wounds, skin infections, and various other dermatological conditions [6,7]. This plant has been reported to possess α-glycosidase inhibitory activity towards carbohydrate hydrolysis and to slow glucose absorption [7]. α-Glucosidase inhibitors have been shown to be effective for people with type 2 diabetes mellitus [8]. According to a previous study by our research group, it was found that the methanol and water extracts of *P. malayana* leaf exhibited noteworthy α-glucosidase inhibitory activity, with IC_50_ values of 2.71 and 6.75 µg/mL, respectively. Palmitic acid, 1,3,5-benzenetriol, β-tocopherol, α-tocopherol, cholesta-7,9(11)-diene-3-ol, 24-epicampesterol, stigmast-5-ene, and monopalmitin were the active anti-diabetic metabolites identified in the extracts [7]. The anti-diabetic efficacy of the aqueous leaf extract was observed in an adult zebrafish model of type 1 diabetes. The extract demonstrated efficacy in reducing and normalizing the blood glucose levels of alloxan-induced zebrafish [9]. This prompts the further optimization of the *P. malayana* leaf extract to augment the inhibitory activity of α-glucosidase. In the pathogenesis of type 2 diabetes, the body’s insulin sensitivity diminishes, thereby inducing insulin resistance, which in turn triggers an inflammatory response. A reciprocal relationship can ensue, wherein heightened inflammation leads to increased insulin resistance, and conversely, increased insulin resistance exacerbates inflammation [10]. The overproduction of reactive species has the potential to exhaust the antioxidant defense system. Therefore, prolonged exposure to elevated levels of reactive oxygen species can result in enduring inflammation and insulin resistance [11,12]. Both the anti-inflammatory and antioxidant properties of OE were evaluated in this study to relate to its anti-diabetic property.

This study employed zebrafish for toxicity testing. The use of zebrafish embryos has gained significance in the fields of toxicology due to some advantages. The optical transparency exhibited by early-life stages of zebrafish enables the examination of visuo-neural circuitry functionality [13]. Zebrafish share 84% homology to gene-related diseases in mammals. Moreover, their use has as low cost, is time effective, and has easy maintenance [14].

Therefore, this study was designed to test the abilities of OE against diabetes, and inflammation and oxidative reactions, to evaluate its toxicity using a zebrafish embryo/larvae model, and to analyze its metabolites.

## 2. Results

### 2.1. Pharmacological Activity Study

The α-glucosidase inhibition (AGI), soybean lipoxygenase inhibitory (SLOXI), 2,2-Diphenyl-1-picrylhydrazyl (DPPH) radical scavenging, and ferric reducing antioxidant power (FRAP) activities of OE are outlined in Table 1. It shows that OE had high AGI activity, SLOXI activity, and antioxidant activities. The strength of the bioactivity is categorized as very strong, strong, moderate, and weak at the IC_50_ values of less than 50, 50–100, 101–150, and more than 150 ppm, respectively [15]. Therefore, OE has very strong AGI and antioxidant activities.

### 2.2. Toxicological Effect Analysis

#### 2.2.1. Morphological, Morbidity, and Mortality Outcomes in Zebrafish (*Danio rerio*) Embryos Treated with OE

Table 2 presents a summary of the morphological defects observed in the zebrafish exposed to OE, as well as the solvent control (1% DMSO in E3 medium), positive control, and E3 medium as the negative control. No observations of morphological changes in the zebrafish group receiving the extract at the concentration of 50 µg/mL were noted, similar to the negative and solvent controls. All morphological defects related to lethality exhibited in the other zebrafish groups. The survival rate was the best in the negative, solvent controls, and zebrafish group treated with OE at a 50 µg/mL concentration; however, the non-survival phenomenon was best observed in the positive and embryo treated with OE groups, especially at concentrations of 100 µg/mL and above.

**Figure 1:**
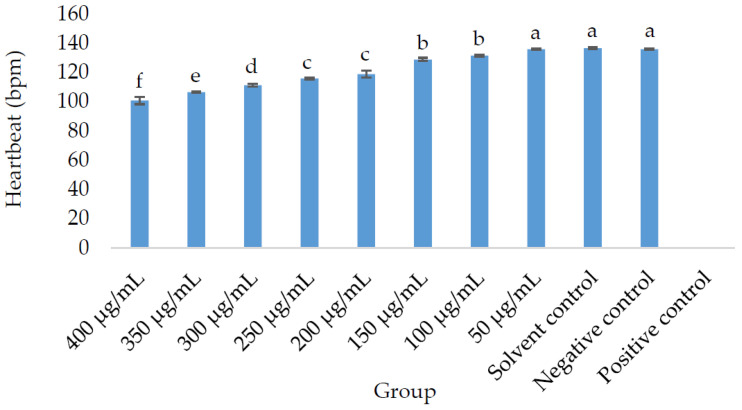
Heartbeats per minute (BPM) of zebrafish embryos or larvae exposed to *P. malayana* leaf optimized extract along with the negative control (E3 medium), 1% DMSO in E3 medium as solvent control, and 4 mg/L of 3,4-dichloroaniline in E3 medium as positive control (). a, b, c, d, e, and f represent significant differences (*p* < 0.05).

The study recorded the beats per minute (BPM) of eight different optimized extracts, in addition to the negative control (E3 medium), 1% DMSO in E3 medium as the solvent control (), and 4 mg/L of 3,4-dichloroaniline in E3 medium as the positive control, as depicted in Figure 1. The heart rate of the 50 µg/mL group compared to the negative control and solvent control did not show a statistically significant difference (*p* > 0.05). However, the heart rate decreased significantly in the embryos fed on higher concentrations of OE, specifically starting at 100 µg/mL and above. Observation of the heartbeat rate in the embryos was not possible due to the occurrence of 100% mortality in the positive control group.

**Figure 2:**
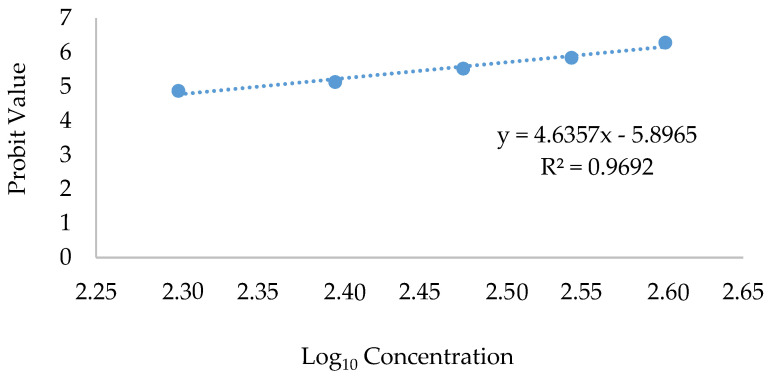
LC_50_ value of *P. malayana* leaf optimized extract logarithm employing probit analysis.

The logarithmic value of 224.29 µg/mL was seen in the probit analysis of the lethal concentration (LC_50_) median (Figure 2). This value was higher than that of our previous study on the non-optimized extract for which the LC_50_ was 37.5 µg/mL for the methanol extract (Nipun et al., 2021) [7]. This finding provides clear evidence that OE exhibits reduced toxicity and can be considered a safer option for continued utilization in the advancement of pharmaceutical drugs.

#### 2.2.2. Teratogenic Effects of Zebrafish Embryo Exposed to OE

The other teratogenic effects, unrelated to the lethal category, observed in the zebrafish are shown in Table 3. This table shows the result of the examination of yolk size. At 24 hpf, a significant reduction (*p* < 0.05) in the yolk size began to be observed in the zebrafish exposed to OE at a concentration of 150 µg/mL. On the other hand, the positive control had a larger yolk size than the negative control.

**Figure 3:**
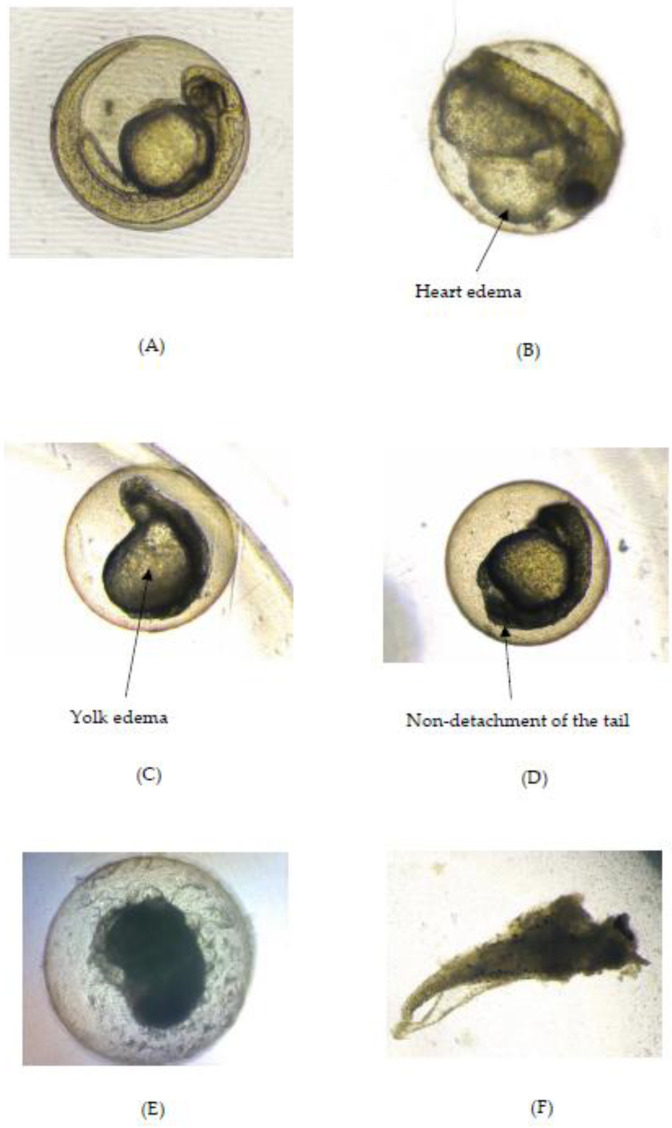
Teratogenic effect in zebrafish embryo or larvae. (**A**) Embryo treated with OE; (**B**) heart edema observed in the positive control; (**C**) yolk edema observed in the positive control; (**D**) non-detachment of the tail observed in the positive control; (**E**) coagulation of the embryo in the positive control; (**F**) coagulation of larvae treated with OE at concentration of 400 µg/mL.

The analysis of the body length of the larvae exposed to OE at all concentrations, except for 50 µg/mL, was impeded by a hatching defect. Nevertheless, the body length of the zebrafish larvae treated with the extract concentration of 50 µg/mL did not significantly differ from the negative control. The body length of the positive control group was significantly shorter (*p* < 0.05) than the negative control group. Similar to the hatching defect, the presence of a pigmentation defect was observed in the zebrafish treated with OE (at a 100 µg/mL concentration), while the heart (Figure 3b) and yolk edema (Figure 3c), as indicated in Table 3, were observed exclusively in the positive control group. The heart and yolk edema were not observed in any of the tested concentrations of OE, or in the negative controls; only the positive control group displayed both defects.

**Figure 4:**
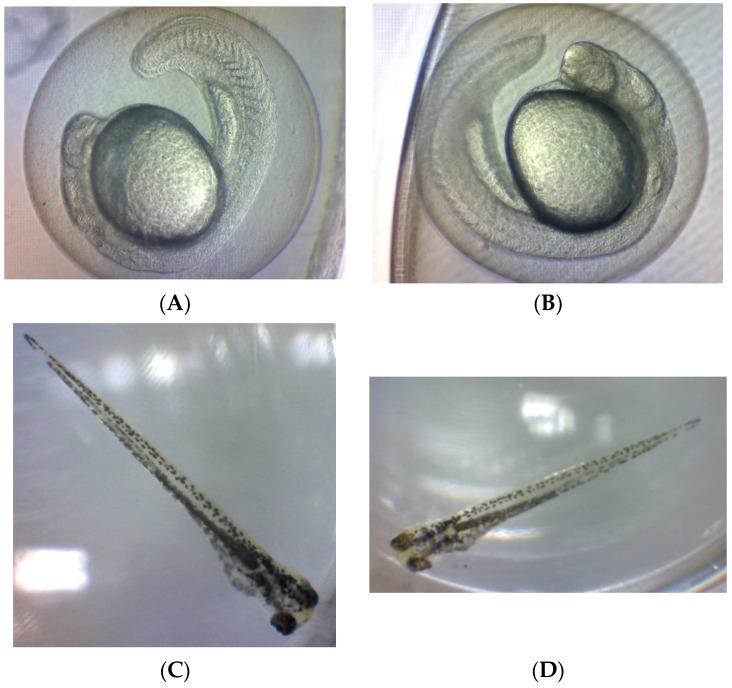
The healthy condition of *Danio rerio* embryo or larvae in negative control and solvent control. (**A**) Embryo in negative control (E3 medium); (**B**) embryo in solvent control (1% DMSO in E3 medium); (**C**) larvae in negative control (E3 medium); (**D**) larvae in solvent control (1% DMSO in E3 medium).

Finally, the observed difference was not significant (*p* > 0.05) in the solvent controls for all observations compared to the negative control, indicating no effect of the solvent on the zebrafish at the applied concentration as shown in Figure 4.

### 2.3. Putative Metabolites of OE Detected by GC-MS and LC-MS

**Figure 5:**
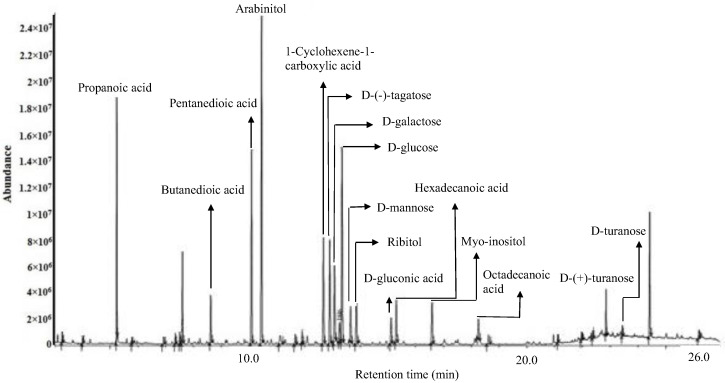
GC−MS chromatogram of the derivatized optimized *P. malayana* leaf extract.

The compounds identified in the optimized extract of *P. malayana* leaves, after undergoing derivatization, were validated by cross-referencing the fragment m/z spectrum of each compound with the mass spectra database provided by the National Institute of Standards and Technology (NIST) 11. The chemical composition of the extracts was partially elucidated through GC-MS analysis following derivatization. Table 4 displays the putative compounds, their retention time, peak area, similarity index (SI), and molecular formula. A variety of phytoconstituents belonging to different categories were identified, including organic acids (propanoic acid, butanedioic acid, pentanedioic acid, 1-cyclohexene-1-carboxylic acid, hexadecanoic acid, D-gluconic acid, and octadecanoic acid), sugar alcohols (arabinitol, ribitol, and myo-inositol), and simple sugars (D-(−)-tagatose, D-galactose, D-mannose, D-glucose, D-(+)-turanose, and D-turanose). The similarity index of all these compounds was equal to or greater than 90%. The putative compounds were identified and labelled at their corresponding peaks in the representative GC-MS chromatogram of the optimized extract (Figure 5).

**Figure 6:**
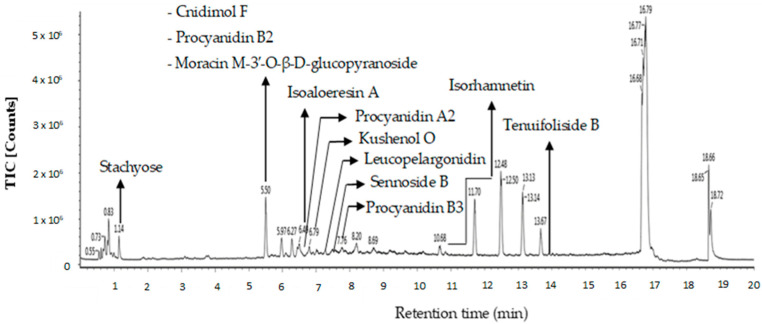
LC−MS chromatogram of OE.

Twelve putative compounds identified in OE of *P. malayana* utilizing the LC-MS spectra are depicted in Figure 6. Several classes of bioactive compounds, including flavonoids, flavonoid glycosides, chromones, tannins, phenylpropanoids, and sugar, were identified. The nomenclature, retention time, observed m/z, neutral mass, mass error, response, adducts, and molecular formula of the putative compounds are presented in Table 5. The compounds that were identified in this study include cnidimol F, procyanidin B2, stachyose, moracin M-3′-O-β-D-glucopyranoside, isoaloeresin A, procyanidin A2, kushenol O, leucopelargonidin, sennoside B, procyanidin B3, isorhamnetin, and tenuifoliside B. The compounds were identified and labeled based on their respective peaks in the OE chromatogram of the liquid chromatography–mass spectrometry (LC-MS) as in Figure 6.

## 3. Discussion

This study builds upon our prior research that demonstrated the potential of the methanol extract of *P. malayana* leaf to exhibit strong anti-diabetic activity [7]. Then, in an effort to increase bioactivity and decrease toxicity, we optimized the extraction of this plant’s leaves. We currently have the trade secret number of TS 2023-01 and this extraction optimization has been registered for the patent filing process. In this study, the in vitro anti-diabetic, anti-inflammatory, and antioxidant properties, and the in vivo toxicity of OE were evaluated.

The AGI-IC_50_ of OE is 2.02 µg/mL. The optimization process successfully resulted in a more potent impact when compared to the previous study, which reported the AGI-IC_50_ of 2.71 µg/mL [7]. In addition, the present study demonstrates the anti-inflammatory impact of OE as per the lipoxygenase inhibition. The IC_50_ value of the extract for this enzyme was found to be 4.92 µg/mL. The present investigation represents the first report on the evaluation of the anti-inflammatory properties of *P. malayana* leaf extract.

α-Glucosidase inhibitors have the ability to slow down the release of D-glucose from oligosaccharides and disaccharides found in complex carbohydrates in our diet. This delay in glucose absorption leads to lower levels of glucose in the blood after a meal and helps to suppress postprandial hyperglycemia [16], while lipoxygenase (LOX) catalyzes the reaction between unsaturated fatty acids, resulting in the production of active lipid metabolites. These metabolites have been implicated in the pathogenesis of several disease conditions including diabetes [17,18,19,20].

OE demonstrated an excellent AGI activity that effectively prevented the absorption of disaccharides. This dual action may contribute to the enhanced anti-adipogenesis and anti-obesity efficacy of the extract, ultimately leading to a significant weight loss. In subsequent periods, it is imperative to ascertain the potential impact of optimized extract on glucose and lipid metabolism in pertinent tissues, including skeletal muscle and the liver [21].

Based on the results obtained from the FRAP and DPPH assays, OE exhibits antioxidant properties. The DPPH inhibitory activity of OE was found to be similar (IC_50_ = 13.08 µg/mL) compared to the results reported in a previous study (IC_50_ = 10.85 µg/mL) [7]. The FRAP assay of OE demonstrated the capability of an antioxidant inhibitor to react with a ferric tripyridyltriazine (Fe^3+^-TPTZ) complex, resulting in the formation of a colored ferrous tripyridyltriazine (Fe^2+^-TPTZ) [22]. Investigations were conducted to encompass a broad spectrum of compounds. The DPPH assay exclusively quantifies hydrophobic antioxidants, while the FRAP assay is designed to measure the antioxidant activity of hydrophilic compounds [23].

A hypothesis has been put forward suggesting an elevation in oxidative stress within individuals with diabetes. The activity of oxygen free radicals has the potential to initiate the process of lipid peroxidation. This, in turn, can stimulate the glycation of proteins, inactivating the enzymes, altering the collagen structure and function, and changing the basement membrane homeostasis. These events together can be followed by a series of complications, both short-term and long-term, ending with the occurrence of diabetes [24].

Several morphological defects were observed in the zebrafish exposed to the lower concentration than the one of LC_50_. It explains the cause of the zebrafish lethality. The present study revealed malformations of the embryo as early as 24 hpf, characterized by the occurrence of hatching defects, particularly at concentrations equal to or exceeding 100 µg/mL. Various degrees of morphological defects were observed in larval zebrafish, including yolk sac definitive, small eyes, and short body lengths. These manifestations can be collectively described as developmental retardation [25]. It can be caused by a lack of energy since energy is widely recognized as a fundamental requirement for living organisms to facilitate various physiological processes, including but not limited to growth, development, locomotion, and reproduction [26,27].

Table 3 displays the embryos’ eye and yolk sizes as well as body length after the administration of various concentrations of OE. The decrease in the embryos’ eye and yolk sizes in a concentration-dependent manner was seen at concentrations of higher than and/or equal to 150 and 100 µg/mL, respectively. The alteration in yolk size has been linked to alterations in the functions of the glomerular filtration barrier [28]. The anomalous growth of the yolk size is suspected to lead to the compromised provision of nutrients to zebrafish embryos [29], while the occurrence of decreased eye size was attributed to the impairment of lipid metabolism and the obstruction of the water permeability barrier on the embryo surface [30].

The analysis of body length was limited to the group with a concentration of 50 µg/mL and negative control groups based on the embryos’ loss of the hatching ability when supplied with concentrations higher than or/and equal to 100 µg/mL of the plant extract. The potential for reduced hatchability of the embryos may be attributed to two factors that are the disruption of the hatching enzyme responsible for the digestion of the chorion and hypoxia [31]. Hypoxia refers to a condition wherein there is an insufficient supply of oxygen at the tissue level, leading to an inability to maintain proper homeostasis [32]. The observed reduction in zebrafish pigment development resulting from the exposure of optimized extract can be attributed to a decrease in tyrosinase activity and a downregulation of gene expression associated to melanogenesis [33,34].

Table 3 shows that the administration of OE onto the embryo or larva resulted in the lack of a heartbeat. The reduction in heartbeats in zebrafish embryos has been observed to be linked to the presence of leaks in the endothelial vessels, often leading to cardiovascular dysfunctions [35,36].

The calculation of dosage is of utmost importance in the context of prescribing medication, as it is necessary to prevent the occurrence of adverse effects. This is particularly significant due to the requirement that the therapeutic dosage be maintained at a level below the LC_50_ value [7]. The toxicological impact of OE was assessed by determining the median lethal concentration (LC_50_) to be 224.29 µg/mL, which is higher than that of the previously reported value of 37.50 µg/mL for the methanol extract of this plant [7]. In accordance with the findings, a therapeutic index of 111.03 was attained, representing a significant increase of eightfold compared to the previously reported value of 13.84 for the methanol extract. This finding suggests that OE is safer and has the potential to be an anti-diabetic agent compared to our previous finding [7].

Gas chromatography–mass spectrometry (GC-MS) was employed for the identification and the analysis of 16 OE putative compounds. These compounds include propanoic acid, butanedioic acid, pentanedioic acid, 1-cyclohexene-1-carboxylic acid, hexadecanoic acid, D-gluconic acid, octadecanoic acid, arabinitol, ribitol, myo-inositol, D-(−)-tagatose, D-galactose, D-mannose, D-glucose, D-(+)-turanose. D-mannose, hexadecanoic acid (also known as palmitic acid), and myo-inositol, which have previously been identified in this plant [7]. The major compounds detected were propanoic acid, butanedioic acid (succinic acid), hexadecanoic acid (palmitic acid), and D-(−)-tagatose. These compounds exhibited a similarity index of 95% or higher using National Institute of Standards and Technology (NIST) Standard Database 11.

The study conducted by Natrus et al. [37] demonstrated that propanoic acid exhibited advantageous properties in mitigating endoplasmic reticulum stress induced by diabetes in the ventromedial hypothalamus of rats. In the study conducted by Al-Lahham and Rezaee [38], it was observed that the application of propanoic acid to human subcutaneous adipose tissue led to a notable decrease in inflammatory indicators such as TNF-α and IP-10, as well as MMP-9 and CD163, the macrophage markers. Propanoic acid is hazardous when supplied to male western albino rats with an escalating risk of hepatic damage [39]. Propanoic acid exhibits significant antioxidant properties, effectively neutralizing reactive oxygen species, including hydroxyl radicals, thereby contributing to detoxification processes. *Desmodium canum*, a plant with recognized medicinal properties, contains succinic acid as its primary hypoglycemic agent. This organic acid demonstrates a hypoglycemic impact after a one-time intake as it effectively causes a blood glucose reduction in Sprague Dawley rats [40]. The study conducted by Chien et al. [41] reported the anti-inflammatory properties of succinic acid derivatives. These derivatives were found to exhibit potential immunomodulatory effects against RAW264.7 macrophage cells [42].

According to Hardy et al. [43], palmitic acid administration resulted in the inhibition of glucose uptake specifically in response to insulin stimulation while having no effect on basal glucose uptake. According to Wu et al. [44], it was observed that palmitic acid can induce the upregulation of inflammatory cytokines in macrophages. These cytokines are known to have a significant impact on the inflammatory mechanisms involved in the development of atherosclerosis. Additionally, it was observed that the alteration in microbiota indirectly led to the induction of endoplasmic reticulum stress and subsequent liver damage in zebrafish. The microbiota containing palmitic acid facilitated the uptake, resulting in increased palmitic acid accumulation in the liver and exacerbated hepatotoxicity in zebrafish [45]. According to previous research, there is evidence suggesting that palmitic acid could potentially serve as a significant risk factor for the development of coronary heart disease [46]. OE at concentrations of 100 µg/mL and higher, exhibited a reduced heartbeat compared to the negative control. This observation could potentially be attributed to the presence of palmitic acid.

The study conducted by Ensor et al. [47] demonstrated that D-tagatose exhibited significant efficacy in reducing HbA1c levels among individuals diagnosed with type 2 diabetes, as compared to the administration of a placebo. The study conducted by Kruger et al. [48] did not observe any evidence suggesting that D-tagatose has the potential to enhance the occurrence of lymphoma cancer cells in mouse L5178Y, regardless of the presence or absence of metabolic activation. Furthermore, the study found no alterations in the micronucleus formation of polychromatic erythrocytes in the bone marrow, leading to the conclusion that D-tagatose does not possess genotoxic properties [48]. Research involving the administration of D-tagatose in Sprague Dawley rats at three different doses (4, 12, and 20 g/kg body weight/day) via gastric intubation showed no toxicity or clinical effects in the rats [49,50]. Therefore, it is possible that D-(−)-tagatose does not contribute to the observed toxicity effect exhibited by OE but expresses a prominent effect on anti-diabetic and anti-inflammatory activities.

According to Mashayekh-Amiri et al. [51], myo-inositol is characterized as a second messenger and an insulin sensitizer, which enhances the maintenance of glucose homeostasis and assumes a significant function in the regulation of glucose. Additionally, it demonstrates anti-inflammatory properties by decreasing the expression of the mRNA of TNF-α [52]. Myo-inositol is a well-established antioxidant molecule [53].

The dystrophic pathology of limb muscles in fukutin-related protein (FKRP)-mutant mice was found to be improved when treated with ribitol, which was associated with focal inflammatory infiltrates [54]. Nevertheless, it was observed that ribitol facilitated a rise in the glutathione peroxidase, catalase, and superoxide dismutase activity. This effect is likely a result of an augmented generation of the superoxide radical [55].

LC-MS detected some putative compounds such as isorhamnetin, isoaloeresin A, moracin M-3′-O-β-D-glucopyranoside, procyanidin B3, and leucopelargonidin. Isorhamnetin is a bioactive molecule that undergoes *O*-methylation and is frequently present in medicinal plants including *Ginkgo biloba*, *Hippophae rhamnoides*, and *Oenanthe javanica* [56]. These flavonoids are beneficial in managing diabetes [57]. The therapeutic effects of oral isorhamnetin were examined on a streptozotocin-induced diabetes model. Experimental animals received 10 mg/kg or 20 mg/kg isorhamnetin for 10 days. Isorhamnetin reduced oxidative stress and hyperglycemia, suggesting an anti-diabetic impact [56]. The anti-diabetic properties of isorhamnetin extend beyond the evidential blood glucose levels’ decline. A similar effect was observed in the red blood cells, the sciatic nerve, and the lenses of the rat model where the sorbitol aggregation was stopped [58,59]. Several studies have shown that it is capable of scavenging DPPH and ABTS radicals, as well as inhibiting lipid peroxidation [60,61,62]. Grdović et al. [63] found that isorhamnetin from *Castanea sativa* may protect against streptozotocin-induced oxidative damage and β-cell apoptosis. The study also found a correlation between observed benefits and the antioxidant activity. Isorhamnetin reduced malondialdehyde (MDA) and intracellular reduced glutathione (GSH) levels while increasing superoxide dismutase (SOD) and catalase (CAT) activity, demonstrating this relationship. The study conducted by Yang et al. [64] provided evidence that isorhamnetin has the potential to mitigate acute lung injury generated by lipopolysaccharide (LPS) through the inhibition of COX-2 expression in male BALB/c mice. Nevertheless, Gong et al. [65] reported this compound’s toxic activity. Dong et al. [66] described isorhamnetin as having been seen to have cytotoxic effects on H9C2 cardiomyocytes. Similarly, Liang et al. [67] discovered that isorhamnetin also demonstrates cytotoxicity towards mouse primary hepatocytes. Furthermore, Zhang et al. [68] found that isorhamnetin promotes DNA damage in HepG2 cells.

Isoaloeresin A is a prominent constituent that is present in Aloe ferox, a plant species known for its abundant anti-inflammatory compounds [69,70]. This plant inhibits the secretion of pro-inflammatory markers and cytokines, which are responsible for inducing significant inflammation that ultimately results in acute respiratory distress [71]. However, there is a lack of evidence pertaining to the potential anti-diabetic, antioxidant, anti-inflammatory capabilities as well as the toxicological effects of this compound.

Moracin M-3′-O-β-D-glucopyranoside was extracted from the leaves of *Morus* insignis and exhibited a significant hypoglycemic effect in streptozotocin-induced hyperglycemic rats [72]. The selection of moracin M-3′-O-β-D-glucopyranoside as a promising agent was based on its established anti-diabetic activities [73]. In addition, it demonstrates significant potential in the therapeutic treatment of inflammatory illnesses. This compound exhibited 100% inhibitory activity against soluble epoxide hydrolase, as evidenced by IC_50_ values of 7.7 μM [74]. However, there is a lack of research investigating the effects of antioxidants on moracin M-3′-O-β-D-glucopyranoside.

Procyanidin B3 demonstrates significant inhibitory activity against glucosidase and possesses antioxidant properties, indicating its potential effectiveness in the prevention and treatment of diabetes and in mitigating oxidative stress [75,76,77,78]. Procyanidin B3 demonstrates inherent antioxidant characteristics and displays significant inhibitory effects on lectin-like oxidized LDL receptor-1, a crucial factor associated with the progression of arteriosclerosis [79]. This compound has an IC_50_ value of 40.1 µg/mL, indicating the inhibition of α-glucosidase [80]. Procyanidin B3 on the other hand displayed an IC_50_ value of 7.9 µg/mL in the DPPH test, demonstrating an antioxidant effect, as well as 34.5 µg/mL in the H_2_O_2_ assay [80]. Procyanidin B3 effectively suppressed the generation of inflammation in human nucleus pulposus cells exposed to LPS by upregulating the inducible nitric oxide synthase and COX-2 expression [81]. The potency of procyanidin B3 in mitigating inflammation generated by 12-O-tetradecanoylphorbol-13-acetate in mouse ears surpassed that of indomethacin and glycyrrhetinic acid, which are commonly employed as anti-inflammatory drugs [82].

Leucopelargonidin extracted from *Pseudarthria viscida* roots increases insulin release in the serum of diabetic rats [83,84]. Cherian et al. [85] found that leucopelargonidin derivatives from *Ficus bengalensis* bark significantly increased β-cell insulin production in vitro. Leucopelargonidin-3-O-α-L rhamnoside dimethoxy ether showed antioxidant activity in hyperlipidemic rats [86]. It was not lethal even at 1.8 g/kg in experimental animals [87].

In summary, the presence of these putative compounds has been found to significantly contribute to the potent anti-diabetic, anti-inflammatory, and antioxidant properties of OE. Myo-inositol, isorhamnetin, and procyanidin B3 demonstrated all three bioactivities.

## 4. Materials and Methods

### 4.1. Materials

#### 4.1.1. Sample and Chemicals

A total of 4 kg of leaves from the plant species *Psychotria malayana* Jack were collected from Cermin Nan Gedang, located in the Sarolangun district of the Jambi province in Indonesia. The leaves were identified by Dr. Shamsul Khamis, a taxonomist affiliated with the University of Putra, Malaysia. The specimen with voucher number (PIIUM008-2) was deposited in the Herbarium at Kulliyyah of Pharmacy, IIUM, Kuantan. Organic solvents of analytical and chromatographic quality (ethanol, methanol, and dimethyl sulfoxide), ascorbic acid, Trolox (6-hydroxy-2,5,7,8-tetramethylchroman-2-carboxylic acid), 2,4,6-Tris(2-pyridyl)-s-triazine (TPTZ), sodium chloride (NaCl), potassium chloride (KCl), calcium chloride dihydrate (CaCl_2_.2H_2_O), and magnesium sulphate heptahydrate (MgSO_4_.7H_2_O) were purchased from Merck (Darmstadt, Germany). α-Glucosidase (Megazyme, Bray, Ireland), ρ-nitrophenyl-ρ-D-glucopyranosidase (PNPG), 2,2-Diphenyl-1-picrylhydrazyl (DPPH), quercetin, phenidone, lipoxygenase, and N-trimethylsilyl-N-methyl trifluoroacetamide (MSTFA) (from Sigma-Aldrich Chemical Co., St. Louis, MO, USA) were also purchased.

#### 4.1.2. Instruments

Instruments included a microplate reader (Dynatech MR5000 TECAN, Tecan Group Ltd., Männedorf, Switzerland), inverted microscope, stereomicroscope, Nikon light microscope (Nikon Corporation, Tokyo, Japan), Dino-eye eyepiece camera, and DanioScope software version 1.1 (Noldus Information Technology, Wageningen, The Netherlands).

#### 4.1.3. Disposable Materials

A flat-bottomed 96-well plate, aluminum foil, micropipette tips, and microcentrifuge tube were used.

### 4.2. Methods

#### 4.2.1. Plant Extract Preparation

The preparation of OE was classified as a trade secret (Reference number: TS 2023-01) registered at International Islamic University Malaysia dated 23 June 2023. The patent filing process was registered at International Islamic University Malaysia (application ID: 3070, dated 24 July 2023). OE was prepared using the response surface methodology (RSM) approach considering three variables: solvent concentration, temperature, and extraction period. The reflux extraction technique, and ethanol as solvent extractor, were employed in the experiment. A total of 18 extracts were produced including one extract validated as an optimized extract.

#### 4.2.2. Pharmacological Activity

##### α-Glucosidase Inhibitory Activity

The AGI activity assay was carried out in accordance with the procedure described by Javadi et al. [88], which resembles the environment of intestinal fluid in the laboratory. Quercetin (1 mg/mL) was used as a standard drug because it is a well-known α-glucosidase enzyme inhibitor, and PNPG (0.3 mg/mL) was used as a substrate. In total, 10 µL of extracts, quercetin, and negative control (DMSO) were accurately placed into a 96-well plate using a micropipette. Amounts of 100 µL of phosphate buffer and 15 µL of α-glucosidase type one from *Saccharomyces cerevisiae* were added to the reaction mixture containing the samples and the control. Meanwhile, the blank was made up of 115 µL of the buffer without enzyme added to the mixture. A total of 75 µL of PNPG was added to each of the test samples and the blank mixture after 5 min at room temperature incubation. After another 15 min of incubation, the process was halted by adding 50 µL of glycine (pH 10) to the mixture. An optical microplate reader (Tecan Nanoquant Infinite M200, Zurich, Switzerland) operating at 405 nm was used to measure the quantity of p-nitrophenol emitted by the PNPG. The absorbances of the stock solution dilutions for both samples and controls were used to calculate the half-maximal inhibitory concentration (IC_50_) using linear regression analysis. The IC_50_ value determination is an insightful indicator of a reactant’s efficacy and potency, as it reflects the quantity of a reactant or inhibitor necessary to lower the reaction by half in each experiment. All determinations were made in triplicate to ensure accuracy.

% α-glucosidase inhibition = [(Acontrol − Asample)/Acontrol] × 100%

where Asample and Acontrol represent the sample and negative control’s absorbance, respectively.

##### Soybean Lipoxygenase Inhibitory (SLOXI) Activity

This study followed Chan et al. [89] with a minor modification. In this experiment, a volume of 150 μL of soybean lipoxygenase (SLOX) solution with a protein concentration of 55.2 ng/mL was mixed with 20 μL of the extract in a 50 mM Tris–HCI buffer at pH 7.4. The buffer also contained dimethyl sulfoxide (DMSO) at a concentration of 32% (*v*/*v*). The mixture was then incubated at a temperature of 25 °C for 5 min. The experimental procedure commenced with the introduction of 50 μL of linoleic acid (40 mM) into the reaction mixture. Subsequently, the mixture was subjected to incubation for a duration of 15 min at a temperature of 25 °C while being shielded from light. The termination of the reaction was achieved by introducing 100 μL of FOX reagent, which consisted of a solution containing 1 M sulfuric acid, xylenol orange, iron (II) sulphate heptahydrate, and methanol. Subsequently, the reaction mixture obtained was subjected to an additional incubation period of 30 min at a temperature of 25 °C. This was carried out to facilitate the formation of the chromophore complex Fe^3+^-XO. The control group was comprised of a mixture containing 150 μL of SLOX and 20 μL of a 50 mM Tris-HCI buffer solution with a pH of 7.4, which also contained DMSO at a concentration of 32% (*v*/*v*). In contrast, the blank group did not contain any linoleic acid. The reaction was halted by the addition of FOX reagent and Tris-HCl buffer. The measurement of absorbance was conducted at a wavelength of 560 nm using a microplate reader (Infinite M200, Seestrasse, Männedorf, Switzerland). The percentage inhibition of the test sample was calculated using the following formula:

% inhibition = [(Acontrol − Asample)/Acontrol] × 100%

where Asample refers to the absorbance of the sample and Acontrol refers to the absorbance of the negative control.

##### 2,2-Diphenyl-1-Picrylhydrazyl (DPPH) Radical Scavenging Activity

The methodology for the microplate antioxidant assay was derived from the 96-well plate assay with some modifications [90,91]. A volume of 50 µL of the diluted sample was combined with 150 µL of a methanol solution containing a 0.2 mM concentration of DPPH. In this experiment, DPPH was substituted with methanol for the sample blank. The positive control was prepared by combining 50 µL of methanol with 150 µL of DPPH, whereas the negative control consisted solely of methanol solvent. Following a period of 30 min in a light-deprived environment at ambient temperature, the measurement of absorbance was conducted at λ = 515 nm using a microplate reader. The standard reference used in the study was ascorbic acid. The percentage of DPPH scavenged was determined by employing the following equation:

% DPPH radical = (1 − [A/B]) × 100%

In this context, A represents the difference between the absorbance of the sample and the absorbance of the blank, while B represents the absorbance of the control.

##### Ferric Reducing Antioxidant Power (FRAP)

For the FRAP assay, a working solution was made by mixing 10 parts of an acetic acid buffer with a pH of 3.6, 1 part of a TPTZ solution, and 1 part of a FeCl_3_ solution. Subsequently, the mixture was subjected to incubation at a temperature of 37 °C. The working solution must be fully utilized within a time frame of 1 to 2 h. The tested sample was combined with 180 μL of the FRAP working solution in a 96-well plate. The plate was shaken for a brief period of time. Subsequently, the plate was subjected to incubation at a temperature of 37 °C for a duration of 15 min under dark conditions. The measurement of absorbance was conducted at λ = 593 nm. The standard used in the experiment was Trolox, while DMSO was employed as the control for blank measurements. The concentration of Trolox was chosen within the range of absorbance values from 0.2 to 0.8 in order to construct a standard curve [92].

#### 4.2.3. Toxicological Study

##### Toxicity Test

The toxic effect of OE was observed in accordance with the OECD [93]. The breeding protocols and upkeep of *Danio rerio* were similarly implemented in accordance with the guidelines [94]. The study was reviewed and commented on by the Institutional Animal Care and Use Committee (I-ACUC) (Reference Number: IACUC 2022-003).

##### Maintenance of Zebrafish (*Danio rerio*)

Adult zebrafish of the albino phenotype were procured and housed at the Central Research and Animal Facility (CREAM) IIUM, located in Kuantan. The fish exhibited no macroscopically observable signs of infection or disease and were not subjected to any pharmaceutical treatments (either acute or prophylactic) for a period of two months prior to spawning. They were housed in aquaria with a water volume of 1 L per fish and a consistent photoperiod of 12–16 h. The filtration system and water quality were able to satisfy all the specifications as outlined in the guideline. The water temperature was consistently maintained within a range of 26 ± 1 °C. The fish were provided with dry flake food and artemia three times per day. To maintain optimal water quality, any surplus food and fecal matter were promptly eliminated one hour after each feeding. The fish holding tanks were situated within a closed system, specifically, a multi-rack aquarium. The zebrafish laboratory at CREAM IIUM was responsible for the upkeep of the multi-rack aquarium system.

##### The Breeding Procedure of *Danio rerio* and Maintenance of Embryos

The experiment utilized synchronized zebrafish eggs of advanced age obtained through a controlled breeding technique. The mature fish of both male and female genders, with a ratio of 2:1, were segregated and permitted to engage in mating behavior exclusively upon the initiation of light exposure. The process of fertilization involved the extraction of eggs from the breeding tanks, their subsequent combination, and the random selection of samples for testing purposes. The eggs underwent a triple washing process to eliminate any debris, using E3 medium that was prepared by dissolving four types of salts in 1 L of distilled water. The salts included 0.292 g of 5.0 mM NaCl, 0.0126 g of 0.17 mM KCl, 0.0486 g of 0.33 mM CaCl_2_.2H_2_O, and 0.0814 g of 0.33 mM MgSO_4_.7H_2_O. Additionally, methylene blue (100 uL) was incorporated into the medium to hinder the growth of fungus [7]. The embryos that were damaged or deceased were extracted by employing a Pasteur pipette that had been modified to have an expanded aperture. The eggs were subsequently transferred to a sterile petri dish containing E3 solution and maintained in an incubator set at a temperature of 26 ± 1 °C. The examination of embryonic growth involved conducting microscopic observations at 6 hpf prior to any sample treatment.

##### Preparation of the Sample and Experimental Procedure

The viable fertilized embryos were transferred to a 96-well plate at 6 hpf using a sterile Pasteur pipette. Every well contained a single embryo. The experiment involved the utilization of 20 eggs for each test concentration of OE, 20 eggs for the positive control (consisting of 4 mg/L of 3,4-dichloroaniline in E3 medium), 20 eggs for the solvent control (comprising 1% DMSO in E3 medium), 20 eggs for the negative control (E3 medium), and 4 eggs as the internal plate control for each plate. Each experimental group was allocated a total volume of 300 uL per well. This volume comprised 150 uL of E3 medium, which was aspirated along with a 6 hpf embryo, and an additional 150 uL of the sample prepared in a 1% DMSO solution. In the present study, the concentrations of OE were determined to be 50, 100, 150, 200, 250, 300, 350, and 400 µg/mL in a solution containing 1% DMSO. The plates were incubated at a temperature of 26 ± 1 °C within the incubator setting of light on (14 h) and light off (10 h) [94].

##### Microscopic Observations

The hatching rate, mortality percentage, and LC_50_ values were observed and calculated for all extracts. Lethality can be assessed through four parameters: embryo coagulation, absence of somite formation, failure of tail detachment, and absence of a heartbeat. The researchers employed probit analysis to ascertain the LC_50_ values in the studies of embryo toxicity tests. The term “lethal concentration 50” (LC_50_) refers to a statistical measure used to quantify the concentration of a toxic substance (expressed in µg/mL or mg/L) that is required to cause mortality in 50% of the animal subjects during a specified testing period. The equation provided was used to calculate the rates of mortality.

Rate of mortality (%) = (Number of dead embryos/Initial number of embryos) × 100%

The provided extract was subjected to further analysis to identify additional irregularities pertaining to the hatching rate, yolk size, eye size, crooked backbone, pigmentation, heart edema, yolk edema, and body length. The embryos were observed using a Nikon light microscope (Nikon Corporation, Tokyo, Japan). The evaluation of survival and sublethal outcomes was conducted throughout the duration of the treatment period, specifically, at 24, 48, 72, and 96 hpf. The cardiac pulsations were captured and visualized using a Dino-eye eyepiece camera and a Nikon light microscope, which facilitated the recording and imaging processes, respectively. The non-invasive DanioScope software version 1.1 (Noldus Information Technology, Wageningen, The Netherlands) was utilized for the analysis of both videos and images.

##### Therapeutic Index

The therapeutic index (TI), also referred to as the therapeutic ratio, is a quantitative measure that compares the concentration of a drug in the bloodstream required to produce a therapeutic effect to toxicity [95]. The TI can be determined by dividing the lethal concentration of a drug required to cause mortality in 50% of the population (LC_50_) by the minimum effective concentration needed to produce a desired effect in 50% of the population (IC_50_), expressed as TI = LC_50_/IC_50_.

#### 4.2.4. GC-MS Analysis

OE was subjected to derivatization analysis prior to GC-MS analysis following the methodology described by Javadi et al. [88], with minor adjustments. In the process of derivatization, a quantity of approximately 25 mg of plant extract was dissolved in 50 µL of pyridine. Before being incubated at 60 °C for a duration of 2 h, a volume of 100 µL of methoxamine hydrochloride solution was introduced into the mixture. Subsequently, 300 µL of N-trimethylsilyl-N-methyl trifluoroacetamide (MSTFA) was added, and the mixture was subjected to an additional incubation period of 30 min at 60 °C. Subsequently, the mixture was subjected to an overnight incubation period at room temperature, specifically maintained at 27 ± 1 °C. The GC-MS system (Agilent 6890, Santa Clara, CA, USA) utilized in this study consisted of a gas chromatograph coupled to a selective mass detector (HP 5973). The GC system was equipped with a capillary column DB-5MS, which had dimensions of 0.25 µm (thickness), 250 µm (diameter), and 30 m (length). OE that had undergone derivatization were introduced into the system using a 2 µL injection in splitless mode. The oven was initially set to a temperature of 85 °C and then gradually increased at a rate of 2 °C per minute until it reached the desired temperature of 315 °C. The oven was maintained at this temperature for a duration of 5 min. The flow rate of the helium carrier gas was adjusted to 0.8 mL/min. The mass scanning parameters encompassed a range of 50 to 550 m/z. Subsequently, the fragment m/z spectra of each compound were cross-referenced with the National Institute of Standards and Technology (NIST) 11 database.

#### 4.2.5. LC-MS Identification

This study employed the methodology utilized by Murugesu et al. [96], with a minor adaptation. The sample was analyzed using an LC-MS-QTOF instrument (Agilent 1290 Infinity and 6550 iFunnel, Santa Clara, CA, USA) equipped with an electrospray interface (ESI) operating in positive ion modes. The preparation of the sample involved the utilization of 250 µL of methanol to dissolve 1 mg of each plant extract. The mixture underwent vortexing and sonication for a duration of 15 min, after which 250 µL of water was added. Following this, the sample was subjected to centrifugation for a duration of 15 min to obtain a supernatant that was visually transparent. The resulting clear supernatant was then transferred into an insert glass vial using syringe filtration. The sample injection was performed using a Phenomenex Kinetex C18 core-shell technology 100 Å (250 mm × 4.6 mm, 5 µm, Torrance, CA, USA) column. The recorded temperature of the column was 27 °C. Prior to sample injection, the column was first rinsed with methanol. A volume of 10 µL of the sample was introduced into the auto-sampling system. The elution of samples was conducted using a gradient system, starting from a mixture of 5% methanol in water with 0.1% formic acid and progressing to absolute methanol over a period of 20 min. Subsequently, the machine was utilized for an additional duration of 10 min, employing absolute methanol as the solvent at a flow rate of 0.7 mL/min. This procedure was conducted for a cumulative period of 30 min. The mass spectrometry (MS/MS) data were acquired within the m/z range of 50 to 1500, using a scanning rate of 1 spectrum per scan. The MS/MS spectra were obtained by applying a collision energy ramp of 35 electron volts (eV). The values assigned to the source parameters were as follows: The temperature of the gas was 200 °C, with a flow rate of 14 L/min. The nebulizer was set at 35 psig. The temperature of the sheath gas was 350 °C, with a flow rate of 11 L/min. The software ACD/Spec Manager v.12.00 (Advanced Chemistry Development, Inc. Toronto, Canada) was utilized for the analysis of the LC-MS-QTOF data. The raw data in the (*.xms) format were transformed into the CDF (*.cdf) format using ACD/Spec manager. The data underwent preprocessing using MZmine software version 2.53 (VTT Technical Research Centre, Oulu, Finland). This preprocessing included baseline correction, peak detection, peak filtering, alignment, smoothing, and gap filling. The preprocessed data were saved in a (*.csv) format.

#### 4.2.6. Statistical Analysis

The data analysis was conducted using Minitab 21 software (Minitab Inc., State College, PA, USA), and all data are presented as mean ± standard deviation (SD). The number of replications (n) for the AGI, SLOXI, and antioxidant assays (DPPH and FRAP) was three. In accordance with the OECD guideline, the sample size for the toxicity study was set at *n* = 20. The significant distinctions between the groups were assessed through the utilization of one-way analysis of variance (ANOVA) in conjunction with a post hoc test, specifically, Tukey’s comparison test. The predetermined levels of significance and confidence were established at *p* < 0.05 and 95%, respectively. There is a significant difference between data points that are represented by distinct letters.

## 5. Conclusions

OE demonstrated a greater level of inhibitory activity against α-glucosidase compared to the prior state of the art. It also displayed high efficacy in the inhibition of soybean lipoxygenase-induced inflammation and antioxidant properties. The results of the toxicity assessment conducted on zebrafish embryos demonstrated that the toxicity of OE was less than the prior state of the art; thus, OE has a higher therapeutic index. This suggests that OE is more potent and is safer for pharmaceutical use. Upon careful examination of all relevant factors pertaining to this invention, it becomes evident that OE holds significant practical value. OE holds theoretical significance in the context of developing medicine and healthcare products for the future management of diabetes mellitus. This discovery establishes a robust foundation for conducting in vivo and clinical trials in the future. However, additional research is necessary to isolate, identify, and quantify the novel bioactive compounds responsible for the anti-diabetic and anti-inflammation activities.

## 6. Patents

The preparation of OE was classified as a trade secret (Reference number: TS 2023-01) registered at International Islamic University Malaysia dated 23 June 2023. The patent filing process was registered at International Islamic University Malaysia (application ID: 3070, dated 24 July 2023).

## Figures and Tables

**Table 1:** α-Glucosidase inhibitory activity (AGI), soybean lipoxygenase inhibitory activity (SLOXI), ferric reducing antioxidant power (FRAP), and DPPH radical scavenging activity of OE.

Sample	α-Glucosidase Inhibitory Activity, IC_50_ (µg/mL)	Soybean Lipoxygenase Inhibitory Activity, IC_50_ (µg/mL)	DPPH Scavenging, IC_50_ (µg/mL)	FRAP (mmol TE/mgDW)
Optimized extract	2.02 ± 0.03 ^a^	4.92 ± 0.12 ^a^	13.08 ± 0.84 ^a^	95.44 ± 0.17
Positive controls	Quercetin: 0.99 ± 0.11 ^b^	Phenidone: 0.93 ± 0.02 ^b^	Ascorbic acid: 2.99 ± 1.07 ^b^	NA

All the findings are displayed as mean ± standard deviation (*n* = 3); NA: not analyzed; Means without shared letters (a and b) are significantly different (*p* < 0.05).

**Table 2:** Zebrafish embryos’ morphological defects and mortality rates (%) in comparison with the E3 medium as negative control, 1% DMSO in E3 medium as solvent control, and 4 mg/L of 3,4-dichloroaniline in E3 medium as positive control.

Extract Concentration (µg/mL)/Control Group	Coagulation ofEmbryo	Non-Detachment of the Tail	Lack of Somite Formation	Lack of Heartbeat	Mortality Rate (%)
400	+	+	+	+	90
350	+	+	+	+	80
300	+	+	+	+	70
250	+	+	+	+	55
200	+	+	+	+	45
150	+	+	+	+	35
100	+	+	+	+	15
50	−	−	−	−	0
Negative Control	−	−	−	−	0
Solvent Control	−	−	−	−	0
Positive Control	+	+	+	+	95

(+): present, (−): not present.

**Table 3:** The parameters of the zebrafish embryos/larvae treated with OE, i.e., heart edema, yolk size, eye size, yolk edema, body length, pigmentation, and hatching.

The Extract Concentration (µg/mL)/Control Group	Yolk Size × 10^5^ (µm^2^) at 24 hpf	Eye Size × 10^5^ (µm^2^) at 96 hpf	Body Length (mm) at 96 hpf	Hatching Defect	Less Pigmentation	Yolk Edema	Heart Edema
400	4.96 ± 0.04 ^f^	0.75 ± 0.02 ^b^	NA	+	+	−	−
350	4.96 ± 0.08 ^f^	0.80 ± 0.04 ^b^	NA	+	+	−	−
300	5.11 ± 0.05 ^def^	0.71 ± 0.04 ^b^	NA	+	+	−	−
250	5.09 ± 0.03 ^ef^	0.76 ± 0.01 ^b^	NA	+	+	−	−
200	5.18 ± 0.05 ^bcde^	0.77 ± 0.03 ^b^	NA	+	+	−	−
150	5.12± 0.05 ^cdef^	0.76 ± 0.01 ^b^	NA	+	+	−	−
100	5.23 ± 0.11 ^bcde^	0.75 ± 0.03 ^b^	NA	+	+	−	−
50	5.28 ± 0.12 ^bcd^	1.04 ± 0.05 ^a^	2.80 ± 0.00 ^a^	_−	−	−	−
Solvent control	5.31± 0.03 ^bc^	1.02 ± 0.02 ^a^	2.80 ± 0.00 ^a^	−	−	−	−
Negative control	5.33 ± 0.02 ^b^	1.05 ± 0.01 ^a^	2.73 ± 0.12 ^a^	−	−	−	−
Positive control	5.63 ± 0.04 ^a^	0.44 ± 0.05 ^c^	2.07 ± 0.12 ^b^	+	+	+	+

(+): present; (−): not present; NA: not analyzed due to hatching defect. The values are presented as mean ± SD, *n* = 20. The means with significant difference of *p* < 0.05: add no letters. Means without a shared letter are significantly different (*p* < 0.05). The larvae exposed to OE at concentrations of 100 µg/mL and higher showed significant data related to the eye size (*p* < 0.05) in comparison with the negative control. In general, the smallest eye size was reported in the positive control group.

**Table 4:** GC-MS identification of OE putative compounds following their derivatization.

Putative Compounds	Retention Time (min)	Area	Similarity Index (SI)	Molecular Formula
Propanoic acid	3.3	255,716	97	C_3_H_6_O_2_
Butanedioic acid	7.4	435,553	96	C_4_H_6_O_4_
Pentanedioic acid	10.1	10,445,703	92	C_5_H_8_O_4_
Arabinitol	10.5	12,476,129	94	C_5_H_12_O_5_
1-cyclohexene-1-carboxylic acid	11.7	548,639	94	C_7_H_10_O_2_
D-(−)-tagatose	12.9	4,801,682	95	C_6_H_12_O_6_
D-galactose	13.3	1,301,519	92	C_6_H_12_O_6_
D-glucose	13.4	8,866,752	94	C_6_H_12_O_6_
D-mannose	13.7	1,987,514	94	C_6_H_12_O_6_
Ribitol	13.9	2,429,392	91	C_5_H_12_O_5_
D-gluconic acid	15.1	1,660,291	92	C_6_H_12_O_7_
Hexadecanoic acid	15.3	1,105,406	96	C_16_H_32_O_2_
Myo-inositol	16.6	2,344,357	94	C_6_H_12_O_6_
Octadecanoic acid	18.6	254,088	94	C_18_H_36_O_2_
D-(+)-turanose	22.8	1,243,470	90	C_12_H_22_O_11_
D-turanose	23.4	325,253	90	C_12_H_22_O_11_

**Table 5:** LC-MS identification of OE putative compounds following their derivatization.

Putative Compound	Observed RT (min)	Observed m/z	Neutral Mass (Da)	Mass Error (ppm)	Response	Adducts	Molecular Formula
Stachyose	1.14	689.21	666.22	0.4	10,432	+Na	C_24_H_42_O_21_
Cnidimol F	5.50	291.0849	290.07904	−4.9	22,880	+H	C_15_H_14_O_6_
Procyanidin B2	5.50	579.1495	578.14243	−0.4	284,385	+H	C_30_H_26_O_12_
Moracin M-3′-O-β-D-glucopyranoside	5.50	427.1013	404.11073	3.2	21,649	+Na	C_20_H_20_O_9_
Isoaloeresin A	6.49	563.1545	540.16316	3.7	32,715	+Na	C_28_H_28_O_11_
Procyanidin A2	6.51	577.134	576.12678	0	5308	+H	C_30_H_24_O_12_
Kushenol O	6.80	601.1317	562.16864	−0.2	10,148	+K	C_27_H_30_O_13_
Leucopelargonidin	7.27	291.0849	290.07904	−4.9	5102	+H	C_15_H_14_O_6_
Sennoside B	7.45	885.1808	862.19564	−4.5	5415	+Na	C_42_H_38_O_20_
Procyanidin B3	7.76	579.1496	578.14243	−0.2	9199	+H	C_30_H_26_O_12_
Isorhamnetin	10.68	317.0644	316.0583	−3.7	9380	+H	C_16_H_12_O_7_
Tenuifoliside B	13.91	691.1852	668.19525	1.1	7009	+Na	C_30_H_36_O_17_

## Data Availability

Data is contained within the article.
